# Combined laryngeal inflammation and trauma mediate long-lasting immunoreactivity response in the brainstem sensory nuclei in the rat

**DOI:** 10.3389/fnint.2012.00097

**Published:** 2012-11-15

**Authors:** Kristina Simonyan, Xin Feng, Victor M. Henriquez, Christy L. Ludlow

**Affiliations:** ^1^Departments of Neurology and Otolaryngology, Mount Sinai School of MedicineNew York, NY, USA; ^2^Laryngeal and Speech Section, Medical Neurology Branch, National Institute of Neurological Disorders and Stroke, National Institutes of HealthBethesda, MD, USA; ^3^Department of Otolaryngology, Wake Forest School of MedicineWinston-Salem, NC, USA; ^4^National Institute of Dental and Craniofacial Research, National Institutes of HealthBethesda, MD, USA; ^5^Department of Communication Sciences and Disorders, James Madison UniversityHarrisonburg, VA, USA

**Keywords:** larynx, inflammation, brainstem, immunoreactivity, rat

## Abstract

Somatosensory feedback from the larynx plays a critical role in regulation of normal upper airway functions, such as breathing, deglutition, and voice production, while altered laryngeal sensory feedback is known to elicit a variety of pathological reflex responses, including persistent coughing, dysphonia, and laryngospasm. Despite its clinical impact, the central mechanisms underlying the development of pathological laryngeal responses remain poorly understood. We examined the effects of persistent vocal fold (VF) inflammation and trauma, as frequent causes of long-lasting modulation of laryngeal sensory feedback, on brainstem immunoreactivity in the rat. Combined VF inflammation and trauma were induced by injection of lipopolysaccharide (LPS) solution and compared to VF trauma alone from injection of vehicle solution and to controls without any VF manipulations. Using a c-fos marker, we found significantly increased Fos-like immunoreactivity (FLI) in the bilateral intermediate/parvicellular reticular formation (IRF/PCRF) with a trend in the left solitary tract nucleus (NTS) only in animals with combined LPS-induced VF inflammation and trauma. Further, FLI in the right NTS was significantly correlated with the severity of LPS-induced VF changes. However, increased brainstem FLI response was not associated with FLI changes in the first-order neurons of the laryngeal afferents located in the nodose and jugular ganglia in either group. Our data indicate that complex VF alterations (i.e., inflammation/trauma vs. trauma alone) may cause prolonged excitability of the brainstem nuclei receiving a direct sensory input from the larynx, which, in turn, may lead to (mal)plastic changes within the laryngeal central sensory control.

## Introduction

Sensory feedback from the larynx is an integral component of the central control of upper airway functions, including breathing, deglutition, and voice production. Alterations of the laryngeal sensory input occur frequently and may be induced by laryngeal inflammation (or laryngitis) due to upper respiratory infection and irritation, voice abuse, laryngopharyngeal reflux, smoking, and vocal fold (VF) trauma (Koufman, 1991; Morrison et al., 1999). Furthermore, treatment of some VF pathologies, such as VF paralysis, atrophy and defects due to laser therapy, with the use of injectable implants (e.g., Teflon, bovine collagen) placed either between the thyroid cartilage and the VF muscle or into the VF muscle directly may also lead to the development of hypersensitivity and long-lasting inflammatory responses (Rubin, 1975; Stein et al., 2000; Cashman et al., 2002). Alterations of the laryngeal sensory feedback often result in a variety of pathological laryngeal reflex responses, such as effortful swallowing, laryngospasm, central apnea, reflexive coughing, and chronic dysphonia (Sasaki and Suzuki, 1976; Loughlin and Koufman, 1996; Nishino et al., 1996; Jafari et al., 2003; Bolser, 2004). Some of these pathological responses in humans may represent a life-threatening condition (e.g., laryngospasm), while others may have a profound effect on persons' quality of life (e.g., chronic dysphonia and coughing).

Development of successful treatment and prevention options for patients with abnormal laryngeal responses due to inflammatory reactions to various causes depends, in part, on better understanding of pathophysiological mechanism(s) underlying the alterations of sensory feedback from the larynx. However, to date, our knowledge remains limited about how the brain processes and responds to ongoing alterations of peripheral laryngeal afferent pathways and how these changes might interfere with the integrated sensorimotor control of upper airway functions.

A recent study has examined the short-term effects of acutely induced laryngeal inflammation and trauma in the rat and reported that enhanced *c-fos* expression may be detected within 6–18 h from insult in the brainstem sensory and motor nuclei, including the solitary tract nucleus (NTS), lateral reticular nucleus and nucleus ambiguus (NA) (Park et al., 2005). Although this study has provided the first evidence of central immunoreactivity changes in response to peripheral laryngeal inflammation, it was not able to differentiate between the separate effects of laryngeal inflammation and trauma, possibly due to a relatively short recovery period (6 or 18 h) and thus an overlapping response and confounding effects of both inflammation and trauma. From the clinical perspective, while this study examined brainstem effects during the acute period of laryngeal inflammation and trauma, persistent laryngeal inflammation is usually a more frequent and complicated condition, leading to chronic changes in upper airway regulation and voice production in humans.

The goal of the present study was to investigate the effects of both long-lasting VF inflammation with trauma and VF trauma alone on immunoreactivity of brainstem nuclei involved in the laryngeal sensorimotor control. We hypothesized that persistent VF inflammation combined with trauma but not VF trauma alone would elicit an immunoreactivity response in the brainstem regions of laryngeal somatosensory control in the rat. Combined inflammation and trauma were induced by injection of lipopolysaccharide (LPS) solution into the rat VF, whereas trauma alone was modeled by needle penetration and injection of vehicle solution into the VF. A control group included animals without any laryngeal manipulations (anesthetic controls). The brainstem immunoreactivity response was assessed by examining several immunoreactivity markers, such as the expressions of neural marker *c-fos* protein, pro-inflammatory cytokine IL-1β, and microglia-specific ionized calcium binding adaptor molecule 1 (Iba-1) in the LPS-, vehicle-injected and anesthetic control groups 72 h after the intervention. The choice of survival period was based on an earlier study, which demonstrated that re-epithelialization and granulation formation in the VF tissue starts at 72 h after laryngeal inflammation and/or injury in the rat (Tateya et al., 2006). Further, to determine the immunoreactivity response in the first-order laryngeal afferent neurons, we examined Fos-like immunoreactivity (FLI) in the nodose and jugular ganglia in LPS- and vehicle-injected animals. To establish whether acute alteration in laryngeal sensory feedback may, in general, lead to an FLI increase in the peripheral ganglia, we additionally examined Fos-expression in the nodose and jugular ganglia of another group of animals in a different setting, which received acute electrical stimulation of the internal branch of the superior laryngeal nerve (iSLN).

## Materials and methods

Thirty-four adult female Lewis (LEW/SsNHsd) rats weighing 225–275 g (Harlan, Indianapolis, IN) were included in the study. Female Lewis rats were used due to the susceptibility of this gender and strain to persistent inflammation (Sternberg et al., 1989, 1990; Tonelli et al., 2003). In the experimental setup, group 1 (*n* = 10) received VF injection of the LPS solution (from *E. coli* serotype 0111:B4, Sigma-Aldrich Co., St. Louis, MO) to induce inflammation combined with trauma from needle penetration. LPS is a bacterial endotoxin associated with gram-negative bacteria, which produces a variety of physiological responses, including inflammatory and immune response modulation (Jacobs, 1981). Group 2 (*n* = 10) received VF injection of saline solution to model trauma from needle penetration alone; group 3 (*n* = 10) did not receive any laryngeal manipulations and served as anesthetic control; and group 4 (*n* = 4) received acute direct electrical stimulation of the iSLN to examine the excitability of the first-order neurons of the laryngeal afferent pathway within the nodose and jugular ganglia without changes in VF tissue integrity. All animals were maintained on a 12-h light/dark cycle and given *ad libitum* access to food and water. All animals received humane care in compliance with the National Institute of Health Guide for the Care and Use of Laboratory Animals (NIH Publications No. 80-23, revised 1996). The study protocol was approved by the Animal Care and Use Committee of the National Institute of Neurological Disorders and Stroke, National Institutes of Health.

### Surgical procedure

Each animal was initially anesthetized with a mixture of 3% isoflurane and 100% oxygen delivered via a ventilatory calibrated anesthetic pump to an induction chamber. The anesthetized animal was removed from the chamber, laid supine on the surgical carriage, and maintained on isoflurane through a head mask. An intravenous (IV) catheter was placed into the tail vein to maintain hydration, and a slow drip of saline with 0.1 ml of heparin solution was administered at a rate 3 ml/kg/h.

In groups 1, 2, and 3, isoflurane delivery was gradually reduced and replaced with 0.3–0.5 ml of propofol solution (10 mg/ml) to maintain anesthesia for 30 min. Group 1 and 2 animals were placed in the surgical chair with their mouth fixed in open position using a custom-designed laryngoscope to visualize the larynx (Inagi et al., 1998). Using a transoral microlaryngoscopic approach (Zeiss, Germany), group 1 animals were injected with a sterile LPS solution (10 μg/5 μl) and group 2 animals received an equivalent volume of sterile saline injection (5 μl). Methylene blue crystals were added to both solutions prior to injection to mark the injection site. All injections were placed into the anterior or middle portion of the right VF using a 10 μl Hamilton syringe. Group 3 animals were maintained under propofol anesthesia for 30 min without any laryngeal manipulations. Animals in all groups were closely observed until full recovery and then returned to their cages in the animal care facility.

All animals in groups 1, 2, and 3 were allowed to survive for 72 h to capture the long-lasting VF tissue changes following inflammation and trauma. All animals were monitored by a veterinarian in the animal care facility during the survival period to detect any behavioral and/or physiological changes due to the experimental procedures. All animals' body weight and core body temperature were measured and recorded twice a day.

After the survival period, animals were again anesthetized with isoflurane as described above and then maintained on alpha-chloralose saline solution (0.7 mg/100 ml) at 8–20 μl/min via the tail IV over a 4-h period to control for unintended Fos expression due to handling of the animals and experimental manipulations. A circulating water blanket was placed underneath the animals to prevent hypothermia. In all animals, core body temperature using rectal temperature gauge, blood oxygenation level, heart rate, and respiratory rate were monitored continuously, and checks for the lack of a withdrawal response to a painful stimulus were conducted regularly at 30 min intervals. At the end of the 4-h period, the rats were deeply anesthetized with IV propofol solution (0.6 ml, 10 mg/ml) and perfused transcardially with phosphate buffer solution (PBS, pH 7.4) followed by 4% paraformaldehyde in 0.1 M PBS (pH 7.4) at the rate of 45 ml/min for 10 min.

Group 4 animals were intubated using an endotracheal tube and maintained under isoflurane anesthesia during exposure of the iSLN. The rats were placed into a stereotaxic frame in the supine position, and the trachea was exposed from the cricoid cartilage to the sternal notch using blunt dissection through a midline incision at the level of the hyoid bone. The endotracheal tube was replaced with a tracheal cannula to maintain the animal under isoflurane anesthesia and to allow the larynx to move freely during stimulation of the iSLN. Bipolar hooked wire electrodes were placed into the thyroarytenoid muscle bilaterally to confirm an electromyographic (EMG) response to stimulation of the iSLN. The right iSLN was exposed and a bipolar stimulating electrode was positioned over the nerve at the level of its entry into the larynx. A grounding electrode was placed in the right thigh of the animal. Isoflurane delivery was then gradually reduced and replaced with alpha-chloralose solution via tail IV (1.4 mg/100 ml) with an initial bolus of 0.2–0.4 ml to maintain anesthetic effect at 8–20 μl/min over a 4-h period (the total dose of 670–6.0 mg/kg). The ventilator connected to the tracheal cannula was used to maintain regular breathing. The rats were kept under quiet conditions with low lighting for 3 h before electrical stimulation of the iSLN was performed. This allowed for the control of *c-fos* expression due to handling of the animal and surgical procedures, which were not relevant to the experimental conditions. During this 3-h period, saline-moistened gauze was placed over the surgical site to maintain tissue hydration, and the stimulating electrode was immersed in warm (37°C) mineral oil. Electrical stimulation of the right iSLN was performed using a 100–500 μA stimulus with a pulse width of 0.2 ms and frequency of 0.5 Hz over a period of 45 min. Recordings of vital signs and the EMG of the thyroarytenoid muscle confirmed elicitation of the laryngeal reflex without affecting cardiac or respiratory function. Following the electrical stimulation of iSLN, the animals were maintained under quiet conditions for 20 min. The animals then received an IV injection of heparin solution (0.1 ml, 5000 units/1 mL) followed by barbiturate overdose and bilateral pneumothoraces. Following euthanasia, the animals were perfused with PBS (pH 7.4) followed by 4% paraformaldehyde in 0.1 M PBS (pH 7.4) at the rate of 45 ml/min.

### Tissue preparation

Following perfusion, the brains, larynges, and bilateral portions of the vagal nerve containing the nodose and jugular ganglia were harvested for tissue processing. The brains and larynges were post-fixed overnight in paraformaldehyde and cryoprotected in 30% sucrose solution at 4°C for 3 days. The nodose and jugular ganglia were first blocked in a 20% gelatin solution to preserve tissue entity and then fixed in 4% paraformaldehyde solution overnight before cryoprotection in 30% sucrose solution at 4°C for 2 days. The brainstem was separated from the spinal cord through the transverse section at the level of the obex and from the forebrain at the level of the rostral periaqueductal gray (PAG), serially sectioned in the stereotaxic frontal plane at 40 μm on a freezing microtome (Zeiss, Germany), and collected into separate wells with 0.1M PBS (pH 7.4) for further immunostaining for Fos protein, pro-inflammatory cytokine IL-1β, microglia-specific ionized calcium binding adaptor molecule 1 (Iba-1), and control Nissl stain.

Brain sections were washed twice in PBS and incubated in 0.1% H_2_O_2_ for 30 min to inhibit endogenous peroxidase activity. Following another three washes in PBS, sections were blocked in 0.4% PBS/Triton X-100 (PBS-T) containing 1.5% normal goat serum for Fos immunostain, 1.5% normal rabbit serum for IL-1β stain, and 1.5% normal goat serum and 1% bovine serum albumin (BSA) for Iba-1 stain, respectively, for 1 h at room temperature. Sections were then incubated with rabbit anti-Fos polyclonal antibody (Ab-5, dilution 1:5000; Calbiochem, San Diego, CA), anti-rat IL-1β antibody (dilution 1:1000; R&D Systems, Minneapolis, MN), and rabbit anti-Iba1 antibody (dilution 1:5000; Wako Chemicals, Richmond, VA), respectively, in PBS-T overnight at 4°C. After three washes in PBS, the sections were incubated in biotinylated anti-rabbit IgG antibody for Fos and Iba-1 immunostains (dilution 1:200, Vector Laboratories, Burlingame, CA), respectively, and in biotinylated anti-goat IgG antibody for IL-1β immunostain (dilution 1:200, Vector Laboratories, Burlingame, CA) for 1 h at room temperature. For the control staining, only normal rabbit IgG was used. Sections were further incubated in Vectastain Elite ABC-kit (Vector Laboratories, Burlingame, CA) for 1 h at room temperature and in the DAB substrate kit solution (Vector Laboratories, Burlingame, CA) for 5 min at room temperature to visualize the bound antibody as a brown reaction product. Sections were then washed and mounted onto chrome-alum-gelatin coated slides. After drying overnight, the control sections were counterstained with cresyl violet. All slides were then dehydrated through an ascending alcohol series and xylene and cover slipped.

The blocked nodose and jugular ganglia were serially sectioned at 20 μm on a freezing microtome (Zeiss, Germany). The sections were processed for Fos immunostain as described above.

The larynges obtained from the animals with LPS and vehicle injections were sectioned at 20 μm and stained with Meyer's hematoxylin and eosin (H&E) for verification of the injection site and evaluation of VF changes due to experimental procedures.

### Quantitative analysis

Data analysis was conducted blinded without prior knowledge of animal group identity. For each of Fos, IL-1β and Iba-1 immunostains, brainstem sections 100 μm apart from each other were examined bilaterally and quantified from the rostral PAG to the caudal medulla in each animal. The brainstem sections were reconstructed using the corresponding Nissl-stained control sections and the stereotactic atlas of the rat brain (Paxinos and Watson, 1998) on an image analysis system (Neurolucida, MicroBrightField, Colchester, VT). Examined brainstem regions included the second-order sensory nuclei of laryngeal afferents, that is the NTS, spinal trigeminal nucleus (Sp5), and intermediate/parvicellular reticular formation (IRF/PCRF) (Altschuler et al., 1989; Patrickson et al., 1991; Travers and Norgren, 1995; Hayakawa et al., 2001); the laryngeal motor nucleus, that is the NA (Gacek, 1975; Yoshida et al., 1982); the higher-order nucleus of laryngeal control, that is the PAG (Ambalavanar et al., 1999); and the non-specific nuclei of the laryngeal control, that is the area postrema (AP) and locus coeruleus (LC) (Ambalavanar et al., 2004). Within the NTS, we examined its medial (NTSm), interstitial (NTSi), and intermediate (NTSim) subnuclei; within the PAG, we examined its lateral (LPAG), dorsomedial (DMPAG), dorsolateral (DLPAG) and ventrolateral (VLPAG) subnuclei. The mean number of immunopositive cells was computed in each of the examined regions on the right (ipsilateral to injection) and left (contralateral to injection) sides of the brainstem (Neurolucida, MicroBrightField, Colchester, VT).

The nodose and jugular ganglia, the sites of the first-order neurons of laryngeal afferents, were examined in vehicle-, LPS-treated, and iSLN-stimulated animals to determine the effects of VF trauma, inflammation, and acute iSLN electrical stimulation, respectively, on FLI in these neurons.

The H&E-stained laryngeal tissue from the LPS- and vehicle-treated animals was examined to quantify the extent and severity of VF changes. The latter was assessed by computing the ratio between the total area of VF damage (in μm^2^) and the total area of the injected VF (in μm^2^), which resulted from the use of methylene blue, on manually outlined laryngeal sections (Neurolucida, MicroBrightField, Colchester, VT). In addition, a scoring system from 0 (none or normal) to 3 (severe or diffuse) (Tsai et al., 2006) was used to estimate epithelial damage and severity of changes in the VF tissue induced by local inflammation and/or trauma.

### Statistical analysis

Because some animals had no response in the same region (marked as zero at quantitative analysis), the data were transformed by adding a constant of one to each cell for each region for all animals before statistical analysis. As the Shapiro–Wilk test found that data were not normally distributed (*W* = 0.811 *p* = 0.019 in the LPS group; *W* = 0.723 *p* = 0.002 in the vehicle group; *W* = 0.841 *p* = 0.045 in the anesthetic group), we used non-parametric tests to assess the statistical differences between the groups while accounting for the variance differences in FLI. We conducted *a priori* Kruskal–Wallis non-parametric analysis to estimate the overall group differences between the LPS-, vehicle-injected, and anesthetic groups, including all examined brainstem structures, at *p* = 0.05. If the group effect was statistically significant on any structure, those structures were further examined using separate post hoc Mann–Whitney *U*-tests to determine the significance of differences in Fos expression between the groups. Because only two brainstem structures (IRF/PCRF and NTSi) were significantly different on the initial Kruskal–Wallis test, the *post-hoc* Mann–Whitney tests were conducted on these structures only with the significance level adjusted to 0.025 to correct for multiple comparisons.

The relationships between FLI in the brain structures that showed differences between the groups (i.e., IRF/PCRF and NTSi) and the severity of VF changes in LPS- and vehicle-treated animals were assessed using Spearman's rank order correlation coefficients at *a priori R* = 0.6 and *p* = 0.05.

Because only spurious IL-1β expression was found in few animals across all experimental groups (2 LPS-treated, 3 vehicle-treated, and 1 anesthetic control) and no positive Iba-1 immunoreactivity was identified in either group, the effects of IL-1β and Iba-1 expressions were not quantified for statistical analyses.

## Results

None of the animals showed distress during the experimental procedures. Recovery during the survival period was uneventful in all animals.

### Larynx

**Figure 1:**
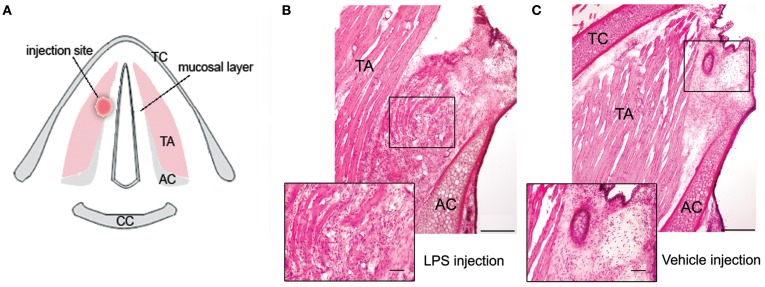
**(A)** Schematic drawing of the transverse section of the larynx in the rat, depicting the site of LPS or vehicle injection in the right vocal fold. **(B)** Photomicrograph of the right vocal fold depicts changes observed following 72 h after LPS injection; the magnified inset shows chronic inflammation and tissue re-organization at the site of LPS injection, characterized by the accumulation of macrophages and lymphocytes in the vocal fold mucosa, disintegrated muscle fibers, and increased vascularization and granulations. **(C)** Photomicrograph of the right vocal fold shows the site of vehicle injection; the magnified inset depicts the absence of formed inflammatory tissue response to trauma from the needle penetration as observed during LPS injection with only few scattered macrophages in the vocal fold tissue surrounding the needle tract. TA, thyroarytenoid muscle; AC, arytenoid cartilage; TC, thyroid cartilage; CC, cricoid cartilage.

In the LPS- and vehicle-treated groups, the location of injection site in the anterior/middle portion of right VF was confirmed by laryngeal microscopy during surgery and postmortem histology of the VF tissue (Figure 1A). While the injection site was well defined immediately after both the LPS and vehicle injections, no visible changes could be seen at the injection site on gross examination of the larynx at the end of the 72-h survival period. The follow-up microscopic examination of VF tissue in LPS-treated animals revealed a picture of chronic inflammation and tissue re-organization in VF epithelium and muscle with the mean severity score of 2.3 (±0.82) affecting on average 29.8% (±18.2%) of the injected VF. These changes were characterized by the accumulation of macrophages and lymphocytes in the VF mucosa, disintegrated muscle fibers, and increased vascularization and granulations (Figure 1B).

In the vehicle-treated group, fewer scattered macrophages were found in the VF epithelium and muscle surrounding the needle tract, and no formed inflammatory tissue response to trauma from the needle penetration was identified (Figure 1C). The mean severity score of VF changes following vehicle injection was 0.7 (±0.67), affecting on average 24.6% (±19.2%) of the VF.

### Brainstem

#### Fos-like immunoreactivity

**Figure 2:**
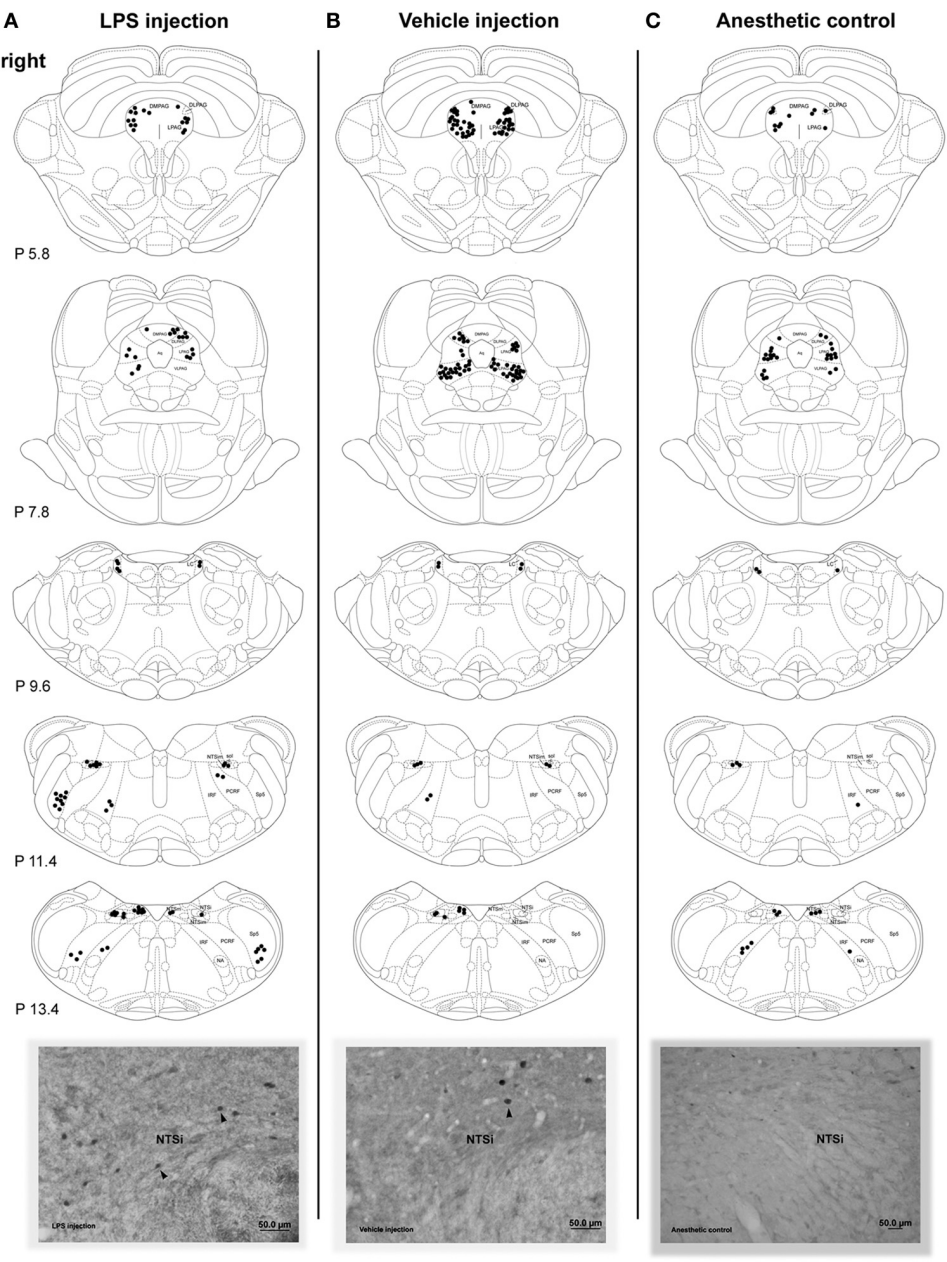
**Distribution of the Fos-positive neurons in the brainstem nuclei 72 h after LPS (A) and vehicle (B) injections into the right vocal fold and in anesthetic control without any laryngeal manipulations (C).** Schematic diagrams depict increased FLI in a single representative animal per respective group. Corresponding photomicrographs below the diagrams show Fos activation in the right NTSi (arrowheads mark Fos-positive nuclei). DLPAG, dorsolateral nucleus of the PAG; DMPAG, dorsomedial nucleus of the PAG; LPAG, lateral nucleus of the PAG; VLPAG, ventrolateral nucleus of the PAG; IRF, intermediate reticular formation; LC, locus coeruleus; NA, nucleus ambiguus; NTS, solitary tract nucleus; NTSi, interstitial nucleus of the NTS; NTSim, intermediate nucleus of the NTS; NTSm, medial nucleus of the NTS; PAG, periaqueductal gray; PCRF, parvicellular reticular formation; sol, solitary tract; Sp5, spinal trigeminal nucleus. Diagrams were based on the atlas of the rat brain (Paxinos and Watson, 1998).

Microscopic evaluation of brainstem sections showed that both groups with LPS-induced VF inflammation/trauma and vehicle-induced VF trauma had bilaterally increased FLI in the NTS (including NTSi, NTSm, NTSim subnuclei), IRF/PCRF, Sp5, PAG (including LPAG, DMPAG, DLPAG, VLPAG subnuclei), LC and AP (Figures 2A,B). Anesthetic controls showed spurious Fos-positive neurons scattered throughout all examined brainstem regions (Figure 2C).

Compared to the vehicle-treated group, Fos expression in the LPS-treated group appeared to be qualitatively stronger in the NTS and IRF/PCRF and weaker in the PAG region. Conversely, no FLI was found in the NA, a site of laryngeal motoneurons, in either experimental or control groups.

**Figure 3:**
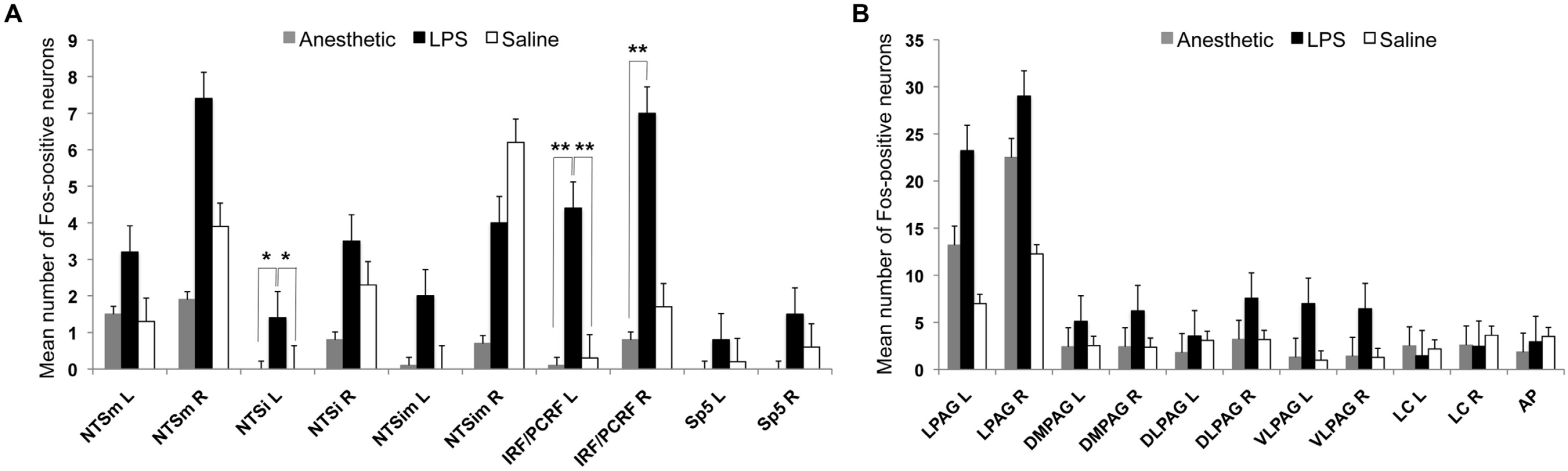
**The bar graphs show the mean number of Fos-positive neurons in the examined brainstem nuclei in the LPS-, vehicle-treated and anesthetic controls. (A)** Double asterisks (^**^) mark significant FLI in the IRF/PCRF nucleus, while single asterisk (^*^) marks a trend in FLI increase in the NTSi nucleus in LPS-treated animals compared to vehicle-treated animals and anesthetic controls. **(B)** No statistically significant differences between these groups were found within the higher-order nuclei of laryngeal afferents (PAG) and non-specific nuclei of laryngeal control (LC and AP). IRF/PCRF, intermediate/parvicellular reticular formation; NTS, solitary tract nucleus; Sp5, spinal trigeminal nucleus; PAG, periaqueductal gray; LC, locus coeruleus; AP, area postrema; NTSi, interstitial nucleus of the NTS; NTSim, intermediate nucleus of the NTS; NTSm, medial nucleus of the NTS; L, left; R, right.

A Kruskal–Wallis test comparing the mean number of Fos-positive neurons in the LPS-, vehicle-treated and anesthetic control groups found statistically significant group differences in the bilateral IRF/PCRF (Kruskal–Wallis test: left χ^2^ = 8.99, *p* = 0.011; right χ^2^ = 7.55, *p* = 0.023) and left NTS_i_ (Kruskal–Wallis test: χ^2^ = 8.89, *p* = 0.012) between the three groups. The follow up Mann–Whitney *U*-tests found that FLI was increased in the bilateral IRF/PCRF (Mann–Whitney test: left IRF/PCRF *U* = 22.5, *p* = 0.015; right IRF/PCRF *U* = 21.0, *p* = 0.013) when comparing the LPS-treated group and anesthetic controls and in the left IRF/PCRF (Mann–Whitney test: *U* = 75.5, *p* = 0.024) when comparing the LPS-treated and vehicle-treated groups. In addition, trends to the FLI increases were found in the left NTS_i_ between the LPS-treated group and anesthetic controls (*U* = 30.0, *p* = 0.030) and between the LPS- and vehicle-treated groups (Mann–Whitney test: *U* = 70.0, *p* = 0.030) (Figure 3A).

There were no significant FLI differences in the higher-order nuclei of laryngeal afferents (i.e., the PAG subnuclei; Kruskal–Wallis test: left χ^2^ = 1.76, *p* = 0.41) or the non-specific nuclei of laryngeal control (i.e., LC, AP; Kruskal–Wallis test: left χ^2^ = 0.69, *p* = 0.73) (Figure 3B) between the three groups. Furthermore, initially (qualitatively) observed FLI increases in the PAG of the vehicle-treated group compared to the LPS-treated group (as depicted on Figure 2) did not reach statistical significance on an exploratory Mann–Whitney test (*U* = 35.0, *p* = 0.068).

**Figure 4:**
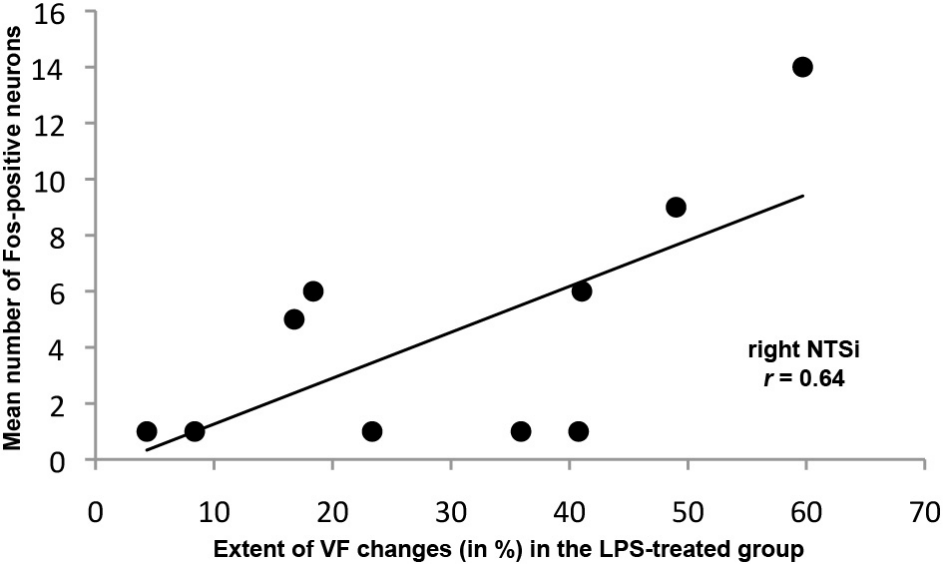
**Significant positive relationship between the mean number of Fos-positive neurons in the right NTSi and the extent of VF changes (in %) following LPS-induced inflammation and trauma**.

The extent of LPS-induced VF changes was positively correlated with FLI in the right NTSi (*R* = 0.64, *p* = 0.044) (Figure 4) while no statistical relationship was found between vehicle-induced VF changes and brainstem FLI (*R* = 0.34, *p* = 0.05).

Taken together, these findings may indicate that combined VF inflammation and trauma but not trauma alone led to potentiation of neural response in the sensory brainstem nuclei of laryngeal afferents, namely in the IRF/PCRF with a trend in the NTSi.

#### IL-1β expression

Only six animals across the three groups (2 with VF inflammation and trauma, 3 with VF trauma alone, and 1 anesthetic control) had scattered positive IL-1β expression in the brainstem regions examined, such as in the NTS and Sp5 in the LPS-treated group, the PAG, LC, Sp5, and NTS in the vehicle-treated group, and the NTSm nucleus in the anesthetic controls. As IL-1β expression was spurious, occurring across all animal groups, it was not quantified for statistical analysis.

### Nodose and jugular ganglia

**Figure 5:**
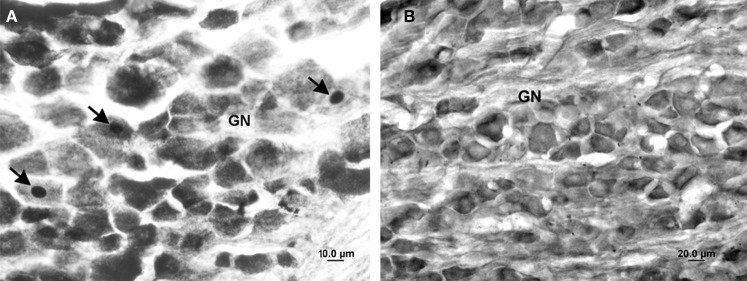
**Photomicrographs of the nodose ganglion in the iSLN-stimulated (A) and LPS-treated (B) animals.** The arrows mark Fos-positive neurons in the iSLN-stimulated animal. No FLI increase was observed in any LPS- or vehicle-treated animal. GN, ganglion nodosum.

No Fos-positive neurons were identified in the nodose or jugular ganglia in the LPS-, vehicle-treated or anesthetic control groups. In contrast, acute electrical stimulation of the iSLN induced scattered FLI in both the nodose and jugular ganglia (Figure 5).

## Discussion

A previous study has shown that acute laryngeal inflammation and trauma produces an FLI response in both the sensory and motor nuclei of the brainstem involved in the laryngeal control (Park et al., 2005). In the present study, we demonstrated that persistent VF inflammation combined with trauma but not trauma alone was associated with an increased brainstem FLI response, which was confined only to the recipient nuclei of laryngeal afferents, particularly the IRF/PCRF and NTS. Fos is a marker of neuronal activation that couples short-term signals elicited by membrane receptor activation with long-term adaptive alterations in cellular function, thus reflecting increased neuronal firing and/or neuronal plasticity changes (Zimmermann and Herdegen, 1994). Our finding of significantly increased Fos expression in the sensory brainstem regions of laryngeal control in the LPS-treated group may point to the ongoing central neuronal hyperexcitability changes following complex modulation of peripheral sensory input from the laryngeal mucosa. These central immunoreactivity changes appeared to have a long-lasting course, even though the peripheral insult by inflammation/trauma was no longer visible during the visual examination of the larynx at the end of survival period. Conversely, the presence of VF trauma alone may have mediated only short-term, transient brainstem FLI changes, which were undetectable by the end of the 72-h survival period. Collectively, our data provide evidence for long-term brainstem sensory but not motor re-organization in response to complex laryngeal alterations due to combined inflammation and trauma. Similar central changes might also be present in patients with chronic alterations of laryngeal sensory feedback, contributing to the development of pathological laryngeal reflex responses, such as effortful swallowing, laryngospasm, central apnea, reflexive coughing, and chronic dysphonia.

### Brainstem sensory recipient nuclei of laryngeal afferents

There is increasing evidence for the importance of IRF/PCRF in the control of laryngeal functions. Earlier studies have identified respiratory and swallowing-related neurons in the medullary reticular formation (Bianchi, 1971; Vibert et al., 1976; Kessler and Jean, 1985; Amri and Car, 1988; Ezure et al., 1993), whereas rapid SLN stimulation has been reported to induce FLI response in different subdivisions of reticular formation, including the IRF/PCRF (Gestreau et al., 1997; Sang and Goyal, 2001; Ambalavanar et al., 2004). Single-unit recording from the IRF/PCRF has been reported to yield vocalization-correlated activity (Larson and Kistler, 1984; Kirzinger and Jurgens, 1991). Due to its direct connections with both sensory (NTS and Sp5) and motor nuclei (n. ambiguus and n. retroambiguus) (Beckstead et al., 1980; Fort et al., 1994), the IRF/PCRF is well positioned to provide interplay between sensory and motor components of the laryngeal control during vocalization, swallowing, and respiration. Our findings of increased FLI following combined VF inflammation and trauma in this region suggest that the IRF/PCRF may, in addition, be involved in the adaptive, and, possibly initially protective, brainstem response to laryngeal inflammation and injury followed by reparative changes in the laryngeal tissue.

Similar to the IRF/PCRF, the NTS is an integrative center, which receives a major proprioceptive input from the VF, terminating mainly in the NTSi and to a lesser degree in the NTSim and NTSm (Kalia and Mesulam, 1980; Nomura and Mizuno, 1983; Hamilton and Norgren, 1984; Lucier et al., 1986; Patrickson et al., 1991; Mrini and Jean, 1995). The neurons in the NTSi are monosynaptically activated by the laryngeal afferents (Jiang and Lipski, 1992), whereas tactile stimulation of the laryngeal mucosa has been reported to elicit Fos expression in the NTSi and NTSm (Tanaka et al., 1995). Our findings of a significant correlation between increased FLI in the NTSi and severity of VF changes following combined inflammation and trauma may indicate a direct involvement of the NTSi in the regulation of VF tissue reorganization due to inflammation and injury.

Although statistically non-significant, Fos expression was observed in the PAG in all three groups: the LPS-, vehicle-injected, and anesthetic controls. The PAG is known to be involved in a wide variety of physiological functions, including vocalization (Larson and Kistler, 1984; Jurgens, 1994; Jurgens and Zwirner, 1996; Ambalavanar et al., 1999) and respiration (Sessle et al., 1981; Davis et al., 1993). A lack of significant Fos expression following laryngeal inflammation and/or trauma compared to anesthetic controls suggests that FLI in the PAG may have either occurred secondary to the plastic changes in the IRF/PCRF and NTS or was triggered by other complex autonomic and sensation processing accompanying laryngeal manipulations, such as nociception (Liebeskind et al., 1973). Alternatively, because Fos marker is biased toward detection of excitation only, it is possible that combined laryngeal inflammation/trauma may have suppressed the excitability of PAG neurons, resulting in fewer Fos-positive neurons in the LPS-treated group compared to the vehicle-treated and control groups.

An interesting feature of significantly increased FLI in the second-order sensory nuclei of laryngeal afferents (i.e., IRF/PCRF and NTS) in the LPS-treated group was the absence of Fos expression in the first-order neurons of laryngeal afferents located in the nodose and jugular ganglia. Previous reports have shown that Fos expression may be observed in these vagal ganglia in acute inflammatory states, such as within 2 h following intraperitoneal and IV injections of bacterial endotoxin as an immune stimulant (Gaykema et al., 1998; Goehler et al., 1998). The follow-up experiments have shown that vagotomy but not sham surgery may abolish Fos expression in the vagal sensory ganglia following intraperitoneal endotoxin injection and strongly attenuated but not eliminated FLI following IV systemic administration (Gaykema et al., 1998). Unfortunately, the only available, to date, report of the effects of acute laryngeal inflammation on brain immunoreactivity did not examine the FLI response in the peripheral vagal ganglia (Park et al., 2005). However, based on the previous studies of acutely induced FLI increase due to inflammation of vagal afferents (Gaykema et al., 1998; Goehler et al., 1998) and our observation of Fos expression in a different experimental setting, such as acute direct electrical stimulation of the iSLN, we may suggest that FLI in the peripheral vagal ganglia might also be present during acute VF inflammation and trauma. On the other hand, the lack of FLI in the vagal ganglia during chronic VF inflammation/trauma may point to a possibility of a more transient neuronal activation in the first-order neurons of laryngeal afferents following chronic alterations of the afferent pathway. Future studies are needed to verify this assumption by a direct comparison of peripheral vagal FLI response between the acute and chronic VF inflammatory states.

In summary, we demonstrated that complex alterations of the sensory input from the larynx due to inflammation combined with trauma but not trauma alone may elicit long-lasting FLI increase in the brainstem nuclei involved in the laryngeal sensory control, possibly by-passing FLI changes in the vagal ganglia neurons. Taken together, this study provides evidence for enhanced central sensory response following laryngeal tissue alterations, which might be the basis for ongoing hypersensitivity changes occurring in patients with chronic laryngeal inflammation. Future research studies may need to explore the effects of different inflammatory agents (e.g., high pH levels, smoking, voice abuse) on VF and brainstem inflammatory responses in order to further dissect the impact of a particular inflammatory agent of VF pathophysiology and its regulation by the central nervous system.

### Conflict of interest statement

The authors declare that the research was conducted in the absence of any commercial or financial relationships that could be construed as a potential conflict of interest.

## Abbreviations

AP: area postrema
DLPAG: dorsolateral nucleus of the periaqueductal gray
DMPAG: dorsomedial nucleus of the periaqueductal gray
IRF: intermediate reticular formation
iSLN: internal branch of the superior laryngeal nerve
LC: locus coeruleus
LPAG: lateral nucleus of the periaqueductal gray
LPS: lipopolysaccharide
NA: nucleus ambiguus
NRA: nucleus retroambiguus
NTS: nucleus of the solitary tract
NTSi: interstitial nucleus of the solitary tract nucleus
NTSim: intermediate nucleus of the solitary tract nucleus
NTSm: medial nucleus of the solitary tract nucleus
PAG: periaqueductal gray
PBS: phosphate buffer solution
PCRF: parvicellular reticular formation
Sp5: spinal trigeminal nucleus
VLPAG: ventrolateral nucleus of the periaqueductal gray.
